# Evaluation of an interaction-skills training for reducing the burden of family caregivers of patients with severe mental illness: a pre-posttest design

**DOI:** 10.1186/s12888-018-1669-z

**Published:** 2018-03-27

**Authors:** Yasmin Gharavi, Barbara Stringer, Adriaan Hoogendoorn, Jan Boogaarts, Bas Van Raaij, Berno Van Meijel

**Affiliations:** 1 GGZ inGeest, Amsterdam, The Netherlands; 2 Research group Recovery and Rehabilitation, GGZ inGeest, Amsterdam, The Netherlands; 3 Department of Psychiatry, Amsterdam Public Health research institute, VU University Medical Center, Amsterdam, The Netherlands; 4 The Mat Training & Education, Amsterdam, The Netherlands; 5 Inholland University of Applied Sciences, Amsterdam, The Netherlands; 6 Parnassia Psychiatric Institute, The Hague, The Netherlands; 7 Academy for Masters in Advanced Nursing Practice, Utrecht, The Netherlands

**Keywords:** Interaction-skills training, IST, Psycho-education, Family caregivers, Self-efficacy, Burden, Severe mental disorders

## Abstract

**Background:**

Family members who care for patients with severe mental illness experience emotional distress and report a higher incidence of mental illness than those in the general population. They report feeling inadequately prepared to provide the necessary practical and emotional support for these patients. The MAT training, an Interaction-Skills Training program (IST) for caregivers, was developed to meet those needs. This study used a single-arm pretest-posttest design to examine the impact of the training on caregivers’ sense of competence (self-efficacy) and burden.

**Methods:**

One hundred family caregivers recruited from three mental health institutions participated in the training. Burden was assessed using the Involvement Evaluation Questionnaire, and self-efficacy using the Self-Efficacy Questionnaire. Analysis of variance with repeated measures was used to investigate whether participation in the training changed the level of family caregivers’ burden and self-efficacy. Pearson’s correlation was used to examine the relationships between self-efficacy and burden.

**Results:**

Our results indicate that, after the training, self-efficacy increased significantly over time (*p* < 0.001) and that burden decreased significantly (*p* < 0.001). However, the results could not demonstrate the expected association between an increase of self-efficacy and decrease of burden**.** Caregivers expressed high appreciation for the training.

**Conclusions:**

After following the IST program, family caregivers of patients with severe mental illness experienced a greater sense of competence and a significant decrease in burden. The training was greatly appreciated and satisfied caregivers’ need to acquire the skills required in complex caregiving situations.

**Trial registration:**

This study was retrospectively registered (14/01/2018) in the ISRCTN registry with study ID ISRCTN44495131.

## Background

A significant proportion of individuals with a severe mental illness (SMI) are unable to manage the demands of daily life. This is mainly caused by their symptoms and the associated impairments of cognitive and social skills. Informal caregivers therefore play an indispensable role in supporting them in their daily activities [1].

As Ostman & Hansson [2] indicated, approximately 90% of individuals with a severe mental illness are supported practically and emotionally by family caregivers on an almost daily basis. But as many caregivers are not properly prepared for a role as informal caregiver, they experience a great deal of psychological strain and a sense of burden [3, 4]. Up to 50% of informal caregivers report significantly more psychological distress and a higher incidence of mental illness than people in the general population [5]. They report in particular feelings of depression, distress, fatigue and trouble sleeping. The burden they experience also intrudes upon their social lives in ways that makes them feel trapped in their caregiving role and isolated from society [6].

Even though previous studies made it clear that patients’ challenging behavior causes caregivers to experience substantial distress and health problems, effective support strategy for reducing the subjective burden are limited [7]. Current interventions to support family caregivers are based mainly on psycho-education [8]. But as caregivers lack the skills and confidence they need to deal effectively with patient’s emotions and behavior [9], they also need practical training to increase their interaction and communication skills [9, 10].

There is limited scientific evidence regarding the effects of broader psychosocial intervention strategies aimed at the reduction of caregivers’ burden. For example, Martire, Lustig, Schulz, Miller and Helgeson [11] described the positive effects of behavioral family therapy on caregiver burden, depression and anxiety. The psychosocial intervention in this therapy targeted the patient’s closest family member or relative. Smeerdijk and colleagues found that the combination of family motivational interventions (MI) and the IST was effective in reducing family members’ worrying and burden [12]. A systematic evaluation by Signe & Elmståhl [13] showed that 90% of family caregivers expressed great satisfaction with respect to psychosocial interventions intended to meet caregiver’s needs. Caregivers seemed to benefit from the network that was created when information and experiences were shared. There is also evidence that caregivers who were exposed to family interventions targeting their caregiver’s needs seemed less disturbed by a patient’s disruptive behavior, because they had learned to attribute it to the illness and not to the patient’s personality [9].

One explanatory variable with respect to the acquisition of these skills and reduction of caregivers’ burden is self-efficacy [14], which concerns people’s beliefs that they can produce the desired effects through their actions [15]. Self-efficacy is seen as a crucial determinant of emotional reactions and burden. Research indicates that lower self-efficacy is associated with greater subjective burden [16].

Late last century, we used available knowledge about burden, self-efficacy and behavioral family interventions to develop the MAT training, being an Interaction Skills Training program (IST) for informal caregivers that would serve the needs of family caregivers. In the present study, we investigated the impact of this training program on the levels of self-efficacy and burden experienced by caregivers caring for a family member with a severe mental illness. We hypothesized that the training was effective in increasing caregivers’ self-efficacy and reducing their burden. Hereinafter, the training will be referred to as “the IST program”.

## Method

### Design

We used a pretest-posttest design to examine the effect of the IST program on self-efficacy and burden, which were measured on three occasions: at T0 (baseline), T1 (after the training) and T2 (three months after termination of the training). The third measurement also included a brief evaluation of the caregivers’ perspective on the training.

### Participants

The training program was offered at three mental health institutions in various parts of the Netherlands. Within these three hospitals, there was an open registration for family members to participate in the training. The training was announced through the mental health professionals, local media, or a brochure. The inclusion criteria were as follows: being a family member of, and caring for, a patient with a severe mental illness, defined as not being free of symptoms, having had the mental illness in the long term (> 2 years), and having serious limitations in personal and social functioning [17]. Informed consent was obtained from all participants and confidentiality was assured. One hundred primary caregivers participated in the training, but, due to incomplete data, only 75 were included in the analyses. Twenty-five participants were excluded from the analysis because of missing data at one of the three measurements T0, T1 or T2.

### The MAT training

The history of MAT started in 1994 when the development of interaction-skills training programs for use in the mental health sector was initiated by Ypsilon, a Dutch advocacy organization committed to family members and close relatives of people with psychosis. These skill-based programs were intended to serve as a tool to improve the interaction and communication between professionals/informal caregivers and patients with psychosis [18].

**Fig. 1:**
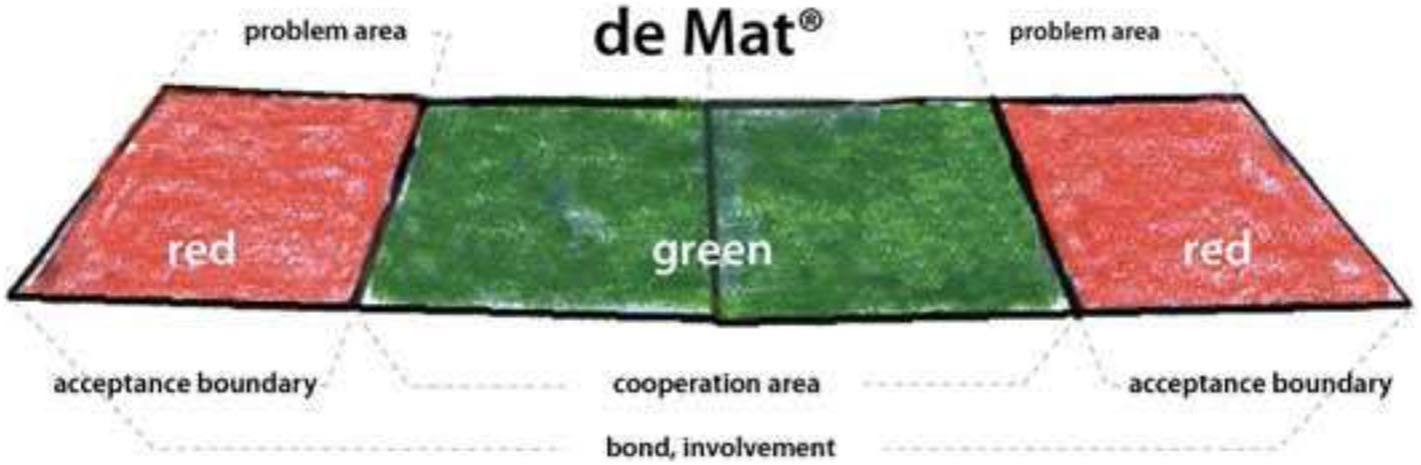
Illustration of the mat

Successful versions of the MAT training have since evolved into the current MAT program, which we used in our study. Its purpose is to provide family caregivers with various interaction skills that can support them in their caregiving role. The MAT is used as a tool to demonstrate through role-play whether or not one is having a problem with another person’s behavior. This is done either by standing on the green section of a mat, which stands for ‘cooperation area’, or on the red section, which stands for ‘problem area’. Either a fellow family member or a MAT trainer takes the position of the patient in the role-plays. In the training, the mat is used to practice several interaction skills that are important in handling difficult confrontations between a family member and a patient, i.e. when one or both of them are standing on the red section of the mat, see Fig. 1.

The complete training comprises seven 3-h sessions over a 10-week period. Our training was taken by a total of eleven groups, with a minimum of eight and a maximum of sixteen participants. It was provided by certified MAT trainers together with trained professionals from a range of disciplines, who also had personal experience of caring for a family member with a severe mental illness. An overview of the training sessions is provided in Table 1. The sessions have a structure that gradually adds new interaction elements to the training with increasing difficulty of the interaction process. The sessions were accompanied by homework assignments with instructions to practice the specific skills that were central in the successive sessions. The participants’ experiences when executing the homework assignments were discussed at the start of the following session.

**Table 1:** Description of the content of the seventraining sessions

Training meeting	Content of meeting
Meeting 1: Goal-oriented interaction	The meeting started with a personal introduction. To improve caregivers’ knowledge of severe mental illness, this was followed by psycho-education and a description of the training program. The participants learned to distinguish between concrete behavior and interpretations. They were also asked to describe specific behaviors of the patient they found hard to deal with. They were then informed about the purpose of the mat in the training. Finally, the participants were asked to formulate their personal learning objectives and were introduced to the homework assignment that would follow each meeting.
Meeting 2: My own life	The homework assignment was discussed. This meeting revolved around challenging the participants to discover how much control they actually had in their own lives. They were asked to write down what they needed in order to feel good, and how much time they needed to perform these activities. If they felt like missing out on these activities, they were asked to draw up a goal plan to serve their needs. Finally, the participants had to come up with three ways of communicating their needs to the patient.
Meeting 3: The communication process	The homework assignment was discussed. The main focus in this meeting, which involved psycho-education, lay on getting a better understanding of transmitting clear messages and listening effectively. The conditions for communicating properly were discussed. Participants practiced their communication skills on the mat.
Meeting 4: My position	After discussion of the homework assignment, the different communication styles and their effect on interaction with the patient were discussed. Participants practiced with the communication styles and with drawing a line when making an issue negotiable. They also learned to make the switch between expressing disagreement properly and listening actively to understand another person’s behavior. To achieve an effective strategy for confrontations when the patient showed resistance, they ended the meeting by practicing the switch.
Meeting 5: Dealing with conflicts	After discussing the previous homework assignment, participants discovered the capacities and incapacities of patients with a severe mental illness with regard to solving a conflict or problem effectively. The participants gained a better understanding of the crucial difference between ‘cannot cooperate’ and ‘will not cooperate’. At the end of the meeting, this psycho-educational form of learning was used on the mat to practice situations in which this problem could arise.
Meeting 6: Working together as a strategy	After discussing the previous homework assignment, participants learned how to cope with the patient when he or she ‘cannot’ cooperate in performing a task, and how to influence the interaction positively. At the end of the meeting, participants were asked to create a management plan to use in teamwork between caregiver, patient and staff as a strategy when contact breaks down.
Meeting 7: Going back home	The last meeting revolved around practicing the skills learnt during the previous six meetings. The learning experiences were translated into real life situations. Finally, the personal learning objectives were discussed and remaining questions were answered.

### Data collection

#### Self-efficacy

Self-efficacy was measured with the Self-Efficacy Questionnaire (SEQ). The SEQ is a questionnaire developed by our research group to measure the extent to which caregivers find themselves competent to interact effectively with a family member with a mental illness. The development process of the SEQ involved the following steps. First, a panel of nine experts, both researchers and MAT trainers, compiled an item pool with items referring to essential competencies of caregivers to effectively interact with the patient with severe mental illness. Next, these items were clustered into meaningful units of caregiver competences. The nine experts participated in four commentary rounds to develop the first version of the questionnaire. This first version of the SEQ was then presented to Ypsilon and five MAT trainers for review with respect to content, formulations and construction. In the last stage, nine caregivers completed the latest version to test its comprehensibility and feasibility. The final version of the SEQ consists of 23 items that are rated on a 4 point Likert scale ranging from ‘1 = probably not’ to ‘4 certainly’. Examples of items are: ‘I think I’m able to make the difference in situations when patient is *not capable* or *not willing* to do something’. Or: ‘I think I’m able to align my own behavior to the patient’s inabilities’. In our sample, the internal consistency of the SEQ was very high, i.e. 0.94. The SEQ is currently subject to further psychometric evaluation, whose results will be published elsewhere.

#### Burden

The Involvement Evaluation Questionnaire (IEQ) measures the degree of subjective burden of caregivers of people with schizophrenia, depression and mixed psychiatric disorders [19]. It covers all domains of burden relevant to caregivers. It was shown to be valid, reliable, easy to understand, not time consuming, and sensitive to changes [20]. With a high degree of internal consistency, ranging from 0.74 to 0.85 for the subscales and 0.90 for all items of the instrument, the IEQ has proven to have satisfactory reliability [19].

The original **IEQ** consists of seven modules. As the second module represents the core module, it was the only module used for this study. The questions concern the burden experienced in the preceding 4 weeks. The items are measured on a 5-point Likert-scale ranging from ‘1 = never’ to ‘5 = (almost) always’. The module contains 31 items which are distributed over the following four subscales: feelings of tension (9 items), which refer to mental or emotional strain; supervision (6 items), which refer to caregiver’s tasks of guarding the patient’s needs; worrying (6 items), which covers feelings of concern and unease; and urging (8 items), which relates to the patient’s activation and motivation to undertake certain activities [20]. The items 28 and 44 are used in more than one scale.

#### Appreciation

To evaluate the appreciation of the training, we developed a short survey with 11 statements referring to the central aims and components of the training (see Table 4). These items are rated on a 4 point Likert scale, ranging from 1 (‘not important’ to 4 (‘very important’). In addition, the respondents were requested to score the overall quality of the training regarding content and trainers (10 point scale: 1 = low; 10 = high).

### Data-analyses

The Analysis of Variance with Repeated Measures (RM-ANOVA) was used to examine the main hypothesis, i.e. whether the training had a significant effect over time on family caregivers’ burden and self-efficacy.

Next, on the assumption that an increase in self-efficacy would result in a decrease in burden, we used linear regression analysis to evaluate the relationship between the increased self-efficacy and decreased burden. Any changes in self-efficacy and burden were calculated using delta scores (endpoint score minus baseline score). Additional exploratory analyses were conducted to investigate the relationship between increased self-efficacy and the different subscales of burden. Finally, we evaluated the relationship between caregivers’ appreciation of the training and any changes of burden and self-efficacy.

The SPSS Statistics program version 20.0 [21] was used for all the analyses. A *p* value of less than 0.05 was considered statistically significant.

## Results

### Sample characteristics

A total of 75 participants were included in the analyses, 25 (33%) of them male and 50 (67%) of them female. Their age range was 25 to 78 years (*Mean* = 55.4, *SD* = 11.1). Forty-eight percent had a lower level of education; 42% had a medium level (bachelor level); and 10% had attended higher education (master level).

### The effect for caregivers’ self-efficacy and burden over time

**Fig. 2:**
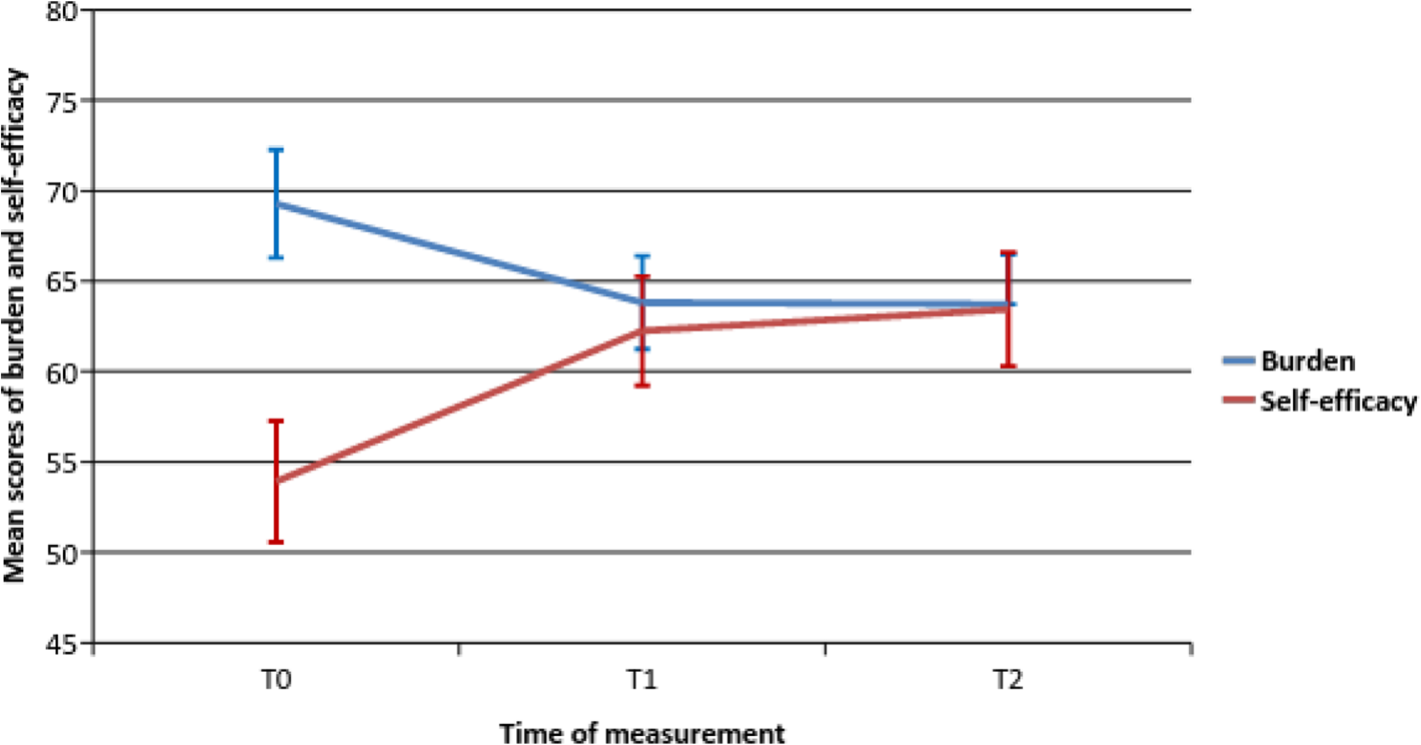
Illustration of the changes in burden and self-efficacy over time. Error bars indicate 95% confidence confidence intervals of the mean

Mauchly’s Test of Sphericity indicated that the assumption of sphericity (i.e., homogeneity of variance) had not been violated for self-efficacy (χ2(2) = 5.34, *p* = 0.069), but had been violated for burden (χ2(2) = 30.74, *p* < 0.001). We therefore corrected degrees of freedom using Lower-Bound estimates of sphericity (ε = 0.74) for burden. The results provide good support for the main hypotheses, showing a significant increase in participants’ self-efficacy after the training (*F*(2,134) = 33.09, *p* < 0.001) and a significant decrease in their burden (*F*(1,70) = 21.37, *p* < 0.001) (Table 2). Figure 2. illustrates these effects. The effect sizes for these analyses (*d* = − 0.71 for self-efficacy, *d* = 0.46 for burden) were classed as large to moderate, in accordance with existing guidelines for interpreting the effect sizes, as offered by Cohen [22].

**Table 2:** Repeated Measures Anova: The effect on self-efficacy and burden over time (T0, T1, T2; N self-efficacy = 68, N burden = 71)

	*Mean (SD)*	*Type III SS*	*df*	*Mean Squared*	*F*	*p*
Self-efficacy						
T0	53.92 (13.91)					
T1	62.24 (12.45)					
T2	63.44 (13.06)					
RM-ANOVA – sphericity assumed:						
within subjects		3652.90	2	1826.45	33.09	< 0.001
error		7395.85	134	55.19		
Burden						
T0	69.29 (12.62)					
T1	63.81 (10.94)					
T2	63.72 (11.55)					
RM-ANOVA – Lower bound corrected:						
within subjects		1448.12	1	1448.12	21.37	< 0.001
error		4743.52	70	67.76

### Correlations between self-efficacy and burden

Pearson’s correlation was used to assess the relationship between the change in self-efficacy and the change in level of burden, including the changes in the subscales of burden (see Table 3). There was no significant correlation between the change in self-efficacy and change in burden (*r* = 0.03, *n* = 70, *p =* 0.78). Neither did additional analyses of the relationship between change in self-efficacy and change in the subscales of burden show any significant correlation (see Table 3).

**Table 3:** Pearson’s correlation: The relationship between change in self-efficacy and change in burden and its subscales (N = 70)

	*r*	95%-Confidence Interval	*p*
Burden	0.03	(−0.20, 0.27)	0.78
Subscales Burden			
Tension	−0.19	(−0.41, 0.06)	0.13
Supervision	0.10	(−0.14, 0.33)	0.40
Worrying	0.07	(−0.17, 0.30)	0.56
Urging	0.15	(−0.09, 0.37)	0.22

### Evaluation of the training

The participants expressed great appreciation for the training: on the 10 point scale, the mean score for training content was 8.4 (*SD* = 0.73); for the trainers it was 8.6 (*SD* = 0.85). There were no significant associations between their appreciation of the training and their decrease in burden (*r*(75) = − 0.102, *p* = 0.384), and their increase in self-efficacy (*r*(75) = 0.092, *p* = 0.432). With regard to appreciation of the different components of the training, the highest average score was for ‘Determining whose problem it is: mine or his/hers’ (*mean* = 3.37, *SD* = 0.56). Table 4 shows the complete list of components, ranked from the highest appreciation to the lowest.

**Table 4:** Appreciation of the components of the training, ranked from highest rated to lowest rated (mean and standard deviations)

1. Determining whose problem it is: his/hers or mine.	3.37	.56
2. Dealing with the handicaps of the person involved.	3.35	.53
3. Insight into the handicaps of the person involved.	3.29	.53
4. Expressing your boundaries.	3.27	.53
5. Recognizing your own boundaries.	3.25	.60
6. Listening attentively: listening to what he/she is trying to tell you.	3.24	.65
7. Listening attentively: actively listening	3.21	.64
8. Practical experience gained by working on the mat.	3.17	.65
9. Formulating your own personal learning objectives.	3.13	.50
10. Guiding your own life.	3.08	.71
11. Confronting the other with his/her behaviour.	3.00	.59

## Discussion

In this study, we found that the caregiver’s level of self-efficacy after the IST had increased, and that their level of burden had decreased. We may conclude that the training program led to meaningful changes in caregiver’s lives. Our results confirm the findings of an earlier study [12] where families who completed the IST reduced burden effectively.

This study did not support Solomon and Draine’s hypothesis that higher self-efficacy is associated with decreased subjective burden [16]. Neither was self-efficacy associated with either of the subscales representing burden. One possible explanation for this is that the great reduction in burden may have been due partly to the supporting network created between caregivers who had to cope with the difficulties, see also Signe & Elmståhl [13]. Another possible explanation is that the follow-up period in our study was too short to assess an association between the increase in self-efficacy and decrease in burden. This effect may develop in later stages when the self-efficacy skills learned have been applied for longer. Caregivers in previous successful versions of the MAT training reported not having enough time to practice the skills they learnt during the training; 75% of them asked for follow-up training [18]. The results of a longer follow-up period should either verify or falsify the hypothesis.

As the evaluation of caregiver’s participation in the training shows, the caregivers regarded the interaction skills training program as very successful, expressing great satisfaction with both its content and the trainers. These results are consistent with the findings of Signe & Elmståhl [13]. With regard to the various training components in our study, caregivers reported great appreciation for ‘determining whose problem it is: mine or his/hers’, ‘dealing with the handicaps’ of the person involved and ‘insight into the handicap’. They also reported feeling more confident about the appropriate things to say, and more competent in ensuring that elaborative conversations did not turn into endless circular discussions. These findings point out the importance of interventions that train caregivers how to effectively manage the caregiver situation. This is consistent with previous research that examined ‘a sense of mastery’ as a caregiving concern [3] and how successful daily interactions between family member and patient were a result of the IST [18].

This study has two main strengths. First, the results of our study regarding self-efficacy and burden, in combination with the caregivers’ great appreciation of the program, provides evidence of the effectiveness of interactive interventions targeting caregiver self-efficacy and burden. As Rose et al. [9] claimed that providing information and support alone is not enough to improve caregivers’ understanding of the mental illness; nor does it increase their sense of competence and confidence in their caregiving ability. Given the current trend in mental health care towards more ambulatory treatment [23] with more caring responsibilities for family members, mental health care should rely less on psycho-educational interventions alone, and more on interaction training programs that include psycho-educational components.

The second strength of our study is that the medium to large effect sizes implicate reliable reduction in burden and improvement of self-efficacy in practical terms for caregivers’ daily life.

Our study also has some limitations. The first was our use of a non-experimental design. Therefore, we could not identify desirable between-group effects by contrasting our results with those of a control group. However, in view of the long-term distress experienced by the family members, spontaneous increase of self-efficacy and decrease of burden over a period of weeks is not very likely [9]. The second limitation is that our findings are based on non-probability sampling. As the caregivers were required to enroll themselves in the training, there may have been selection bias. Similarly, the fact that caregivers were informed about and recruited for the training in various ways may have affected their motivation for signing up for and participating in the training. The third limitation was the relatively small sample size caused by incomplete data; roughly a quarter of our participants were excluded from analyses. Fourth, as there is no follow-up data, we cannot examine the effect over a longer period. Finally, although SEQ showed high face validity, good feasibility and high internal consistancy, further examination of the psychometric properties of the Self-Efficacy Questionnaire (SEQ) is necessary. This psychometric research is currently in progress.

## Conclusions

The main purpose of the IST program is to train caregivers in using effective communication and interactive skills when dealing with patients who frequently behave disruptively. After taking the IST program, family caregivers of patients with severe mental illness experienced a greater sense of competence (self-efficacy) and a significant decrease in burden. The training, combining psycho-education and interactive skills, was greatly appreciated, and satisfied the caregivers’ need to acquire the skills they required in complex caregiving situations.

## Abbreviations

IST: Interaction-Skills Training
MI: Motivational Interventions
